# Instrumental Activities of Daily Living Scales to Detect Cognitive Impairment and Dementia in Low- and Middle-Income Countries: A Systematic Review

**DOI:** 10.3233/JAD-210532

**Published:** 2021-08-31

**Authors:** Heather Yemm, Dame Louise Robinson, Stella-Maria Paddick, Catherine Dotchin, Michaela Louise Goodson, Alla Narytnyk, Marie Poole, Ríona Mc Ardle

**Affiliations:** a Population Health Sciences Institute, Newcastle University, Newcastle Upon Tyne, UK; b Helen McArdle Nursing and Care Research Institute, Faculty of Health Sciences and Wellbeing, University of Sunderland, Sunderland, UK; c Gateshead Health NHS Foundation Trust, Gateshead, UK; d Northumbria Healthcare NHS Foundation Trust, Tyne and Wear, UK; e Medical Research Department, Faculty of Medical Sciences, Newcastle University Medicine, Iskandar Puteri, Malaysia; f Translational and Clinical Research Institute, Newcastle University, Newcastle Upon Tyne, UK

**Keywords:** Activities of daily living, cognitive dysfunction, cross-cultural comparison, dementia, developing countries, diagnosis, functional status

## Abstract

**Background:**

The largest proportion of people with dementia worldwide live in low- and middle- income countries (LMICs), with dementia prevalence continuing to rise. Assessment and diagnosis of dementia involves identifying the impact of cognitive decline on function, usually measured by instrumental activities of daily living (IADLs).

**Objective:**

This review aimed to identify IADL measures which are specifically developed, validated, or adapted for use in LMICs to guide selection of such tools.

**Methods:**

A systematic search was conducted (fourteen databases) up to April 2020. Only studies reporting on development, validation, or adaptation of IADL measures for dementia or cognitive impairment among older adults (aged over 50) in LMICs were included. The QUADAS 2 was used to assess quality of diagnostic accuracy studies.

**Results:**

22 papers met inclusion criteria; identifying 19 discrete IADL tools across 11 LMICs. These were either translated from IADL measures used in high-income countries (*n* = 6), translated and adapted for cultural differences (*n* = 6), or newly developed for target LMIC populations (*n* = 7). Seven measures were investigated in multiple studies; overall quality of diagnostic accuracy was moderate to good.

**Conclusion:**

Reliability, validity, and accuracy of IADL measures for supporting dementia diagnosis within LMICs was reported. Key components to consider when selecting an IADL tool for such settings were highlighted, including choosing culturally appropriate, time-efficient tools that account for gender- and literacy-bias, and can be conducted by any volunteer with appropriate training. There is a need for greater technical and external validation of IADL tools across different regions, countries, populations, and cultures.

## INTRODUCTION

It is estimated that 54 million people are living with dementia globally [1], with numbers set to rise to 75 million by 2030 [2]. Two-thirds of dementia cases occur in low- and middle-income countries (LMICs) [1, 3], yet less than 10% of people with dementia in LMICS receive a diagnosis [1]. This highlights the difficulty in accurately assessing prevalence of dementia globally and leads to difficulties in accessing appropriate care in LMICs. Dementia is a progressive neurodegenerative condition characterized by decline of cognitive performance across multiple cognitive domains, which impacts an individual’s ability to carry out activities of daily living (ADLs) [4]. There are a number of reasons for the low rates of dementia diagnosis in LMICs, including stigmatization, lack of funding and resources for health and social care, variations in assessment and characterization of dementia, and cultural differences regarding the expectation of older adults within society which contributes to low recognition of dementia by family members and society as a whole [2, 3]. Accurate and timely diagnosis of dementia is vital to appropriately treat and manage the disease, educate carers about the condition, and to ensure that people with dementia from LMICs are represented within global dementia research. As such, it is recommended that valid and accurate tools are developed to support dementia screening in LMICs, which are appropriate for variations in culture, education, and language [3].

Subtle cognitive impairments occur years before formal diagnosis of dementia and can manifest through increasing impairments in ADLs [5]. ADLs refer to everyday activities which are associated with functional independence and are a fundamental part of dementia diagnosis [4]. Clinically, they can be separated into more cognitively-driven activities known as instrumental ADLs (IADLs; e.g., shopping, financial management), and more procedural activities known as basic ADLs (BADLs; e.g., eating, bathing) [5]. While difficulties in BADLs tend to occur in later stages of dementia, impairments in IADLs may become increasingly apparent early in the disease course prior to formal diagnosis and reflect the onset of cognitive decline [6]. As such, IADL assessments are recommended as simple and effective screening tools for dementia in LMICs [3].

Multiple questionnaires have been developed to assess IADLs in dementia [7]; however, most are targeted at high-income Western countries and may be culturally-inappropriate for use in LMICs due to different age- and gender-roles, literacy rates and geographical variations [3]. For example, in certain countries there are cultural expectations that younger family members will manage household and financial matters while older adults play a more social role within the community [8]. Therefore, IADL tools with a significant focus on financial management or household chores may not be suitable, while tools which are weighted to social activities, such as presiding over ceremonies or following local affairs, could better reflect cognitive decline. Additionally, some LMICs have unique activities that reflect discrete cultural practices, and which would be considered IADLs (e.g., tying a sari) while their equivalent in Western culture would be characterized as BADLs (e.g., getting dressed). When choosing an IADL assessment to support dementia screening in LMICs, it is important to consider if the tool is culturally-appropriate for the target population in order to maximize the efficacy and accuracy of its use for dementia diagnosis [3]. Therefore, this review aims to support researchers and clinicians in selecting culturally appropriate IADL tools by 1) identifying IADL tools that have been developed or adapted for use in LMICs and 2) reporting how reliable, valid, and accurate these tools are for identifying dementia.

## METHODS

### Identification of studies

#### Search terms and databases

Searches were conducted across fourteen databases, including databases of LMIC-based literature to maximize the opportunity to locate studies involving LMIC populations. The databases searched were: 3ie, AIM, African Journals Online, CINAHL, Eldis, Embase, KCI, LILACS, MedCarib, MEDLINE, PsycInfo, RSCI, SciELO, and World Bank. Search results were limited to studies conducted prior to April 2020 (the date searching commenced) with no lower date limit. To identify studies from LMICs, the Cochrane filter for LMICs was used in databases where this was possible. A list of all countries listed as low-, lower middle-, or upper middle-income as of April 2020 was also obtained from the World Bank Database. Combinations of the search terms described in the Supplementary Material were searched across the databases. This review was pre-registered on PROSPERO (Reference: CRD42018107882).

#### Inclusion criteria

Inclusion criteria were as follows:

1. The study assessed IADLs in older adults aged 50 years or older who had been given a diagnosis of, or were being assessed for, dementia or cognitive impairment.
2. The study was conducted in an LMIC setting, as defined by the World Bank country classification by income database as of April 2020.
3. The study reported at least one of the following:
  a. The validity and reliability of the IADL measure
  b. The sensitivity and specificity of the IADL measure
  c. Positive and/or negative predictive value of the IADL measure
  d. Comparison with a previously validated IADL measure

#### Exclusion criteria

Studies were excluded if they focused on IADL assessments in populations other than those living with dementia or cognitive impairment, as were studies which only involved populations from high-income countries. Studies which did not report any statistical assessments of the diagnostic accuracy or validity of the IADL measure were also excluded. Finally, studies which were not available in English language were excluded due to a lack of resources available for translation.

#### Selection process

**Fig. 1:**
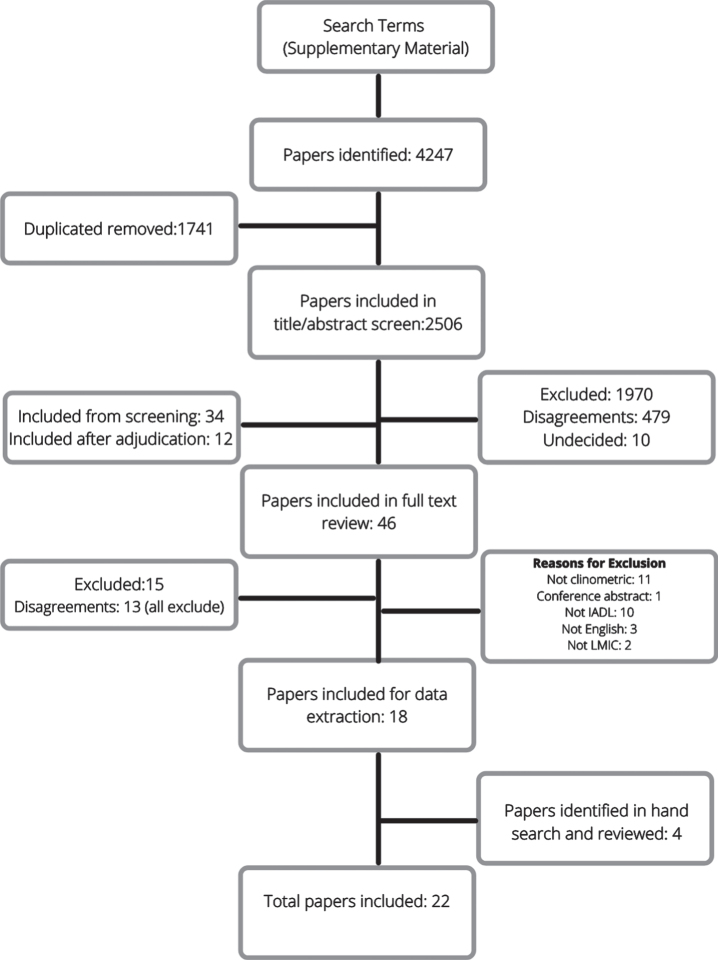
Flowchart of the screening and eligibility evaluation for studies included in the review.

Results from all searches were imported into Microsoft Excel to assist with screening against the inclusion and exclusion criteria. All titles and abstracts were screened by four reviewers (RMA, HY, MG, AN) according to the inclusion criteria. Any discrepancies were referred to an adjudicator to obtain a consensus view. Full text versions of articles that met the inclusion criteria were obtained and each were assessed for final inclusion by two reviewers (from RMA (all texts; *n* = 44), HY (*n* = 5), MP (*n* = 10), MG (*n* = 10), SMP (*n* = 9), AN (*n* = 10)) with discrepancies referred to an adjudicator who had not previously reviewed the specific text (CD (*n* = 12)). Eligibility of identified articles was recorded at every stage to document the review process. Duplicates were identified and removed prior to commencing the screening process. A hand search of reference lists of included studies was also conducted to identify any studies which had not been detected in the search process (HY, CD; see Fig. 1 for further details).

### Data analysis

#### Data extraction

Data were extracted from all eligible articles, with key measures of interest as follows: 1) LMIC country involved; 2) setting (urban/rural, clinic/community/care); 3) type of IADL tools (translated, translated, and adapted, newly developed for target population); 4) criteria used to characterize cognitive impairment/dementia; 5) domains included in the IADL tool (basic, instrumental, advanced); 6) scoring of IADL tool; and 7) clinometric properties of IADL tool (i.e., reliability, validity, accuracy).

#### Interpretation of data

Data was synthesized according to the type of IADL tool that each study employed, i.e., translated, translated and adapted, and newly developed for a target population. This approach was determined after reviewing all studies included in this review. Translated tools refer to IADL tools which were used and/or validated in another country and language, and which were directly translated into a target language (e.g., English to Portuguese). Translated and adapted tools refer to IADL tools which were used and/or validated in another country and language, and which were translated into a target language using a cross-cultural approach, such as making adaptions for terminology or changing items to ensure appropriateness for the target culture. Tools which were newly developed for a target population refers to IADL tools which were developed specifically for the population being studied, usually through consensus processes to ensure that items included in the IADL tool were appropriate and relevant to the culture (e.g., inclusion of “tying lower garments appropriately” in Indian populations).

All studies included in this review reported reliability (internal consistency (e.g., Cronbach’s alpha), test-retest, inter-rater (e.g., ICCs, Pearson/Spearman correlations)), validity (concurrent (e.g., correlations), construct (e.g., correlations), convergent (e.g., correlations), discriminative (e.g., between-group comparisons)), and/or diagnostic accuracy (criterion validity, sensitivity, specificity, positive/negative predictive values, area under the curve (AUC)). Therefore, the current review examined these three types of reliability, four types of validity, and the range of diagnostic accuracy measures. IADL tools which were assessed in multiple studies were highlighted in the results and data were synthesized to provide a comprehensive overview of the evidence.

#### Quality assessment

The Quality Assessment of Diagnostic Accuracy Studies version 2 (QUADAS-2) tool [9] was used to evaluate the quality of included studies. This measure assesses four key domains: 1) method of participant selection; 2) index test use and interpretation; 3) reference standard use and interpretation; and 4) flow and timing of tests. Some of the included articles were not diagnostic accuracy studies and so it was not possible to use the QUADAS-2 to fully assess these as certain domains were not covered. Two reviewers (RMA and SMP) determined quality of all diagnostic accuracy studies in a blinded assessment. Disagreements were settled through consensus.

## RESULTS

### Search yield

The search yielded 4,247 articles, of which 1,741 were duplicates and removed. Following title and abstract search, 46 full texts were obtained and assessed for eligibility, of which 28 were excluded (Fig. 1). An additional four articles were identified via a hand search of reference lists of included studies. In total, 22 studies were included in this review.

### Study characteristics

**Fig. 2:**
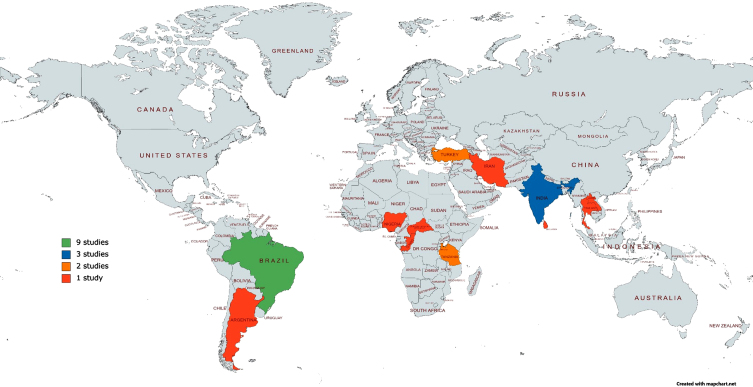
Heat map of locations for research into the development, adaption, and validation of assessments for instrumental activities of daily living to support dementia diagnosis in low-middle income countries.

The characteristics of the 22 eligible studies are summarized in Table 1. All articles were published between 1999–2019. Only one study carried out longitudinal analysis [10]. In order of quantity, countries represented by this review include Brazil (41%; *n* = 9), India (13%; *n* = 3), Turkey (9%; *n* = 2), Tanzania (9%; *n* = 2), Argentina (5%; *n* = 1), Nigeria (5%; *n* = 1), Republic of Congo (5%; *n* = 1), Central African Republic (5%; *n* = 1), Iran (5%; *n* = 1), Sri Lanka (5%; *n* = 1), and Thailand (5%; *n* = 1) (Fig. 2). The sample size ranged from 40–632 participants across studies. Additionally, 82% of studies reported > 50% of participants as female. Prevalence of cognitive impairment in the sample ranged from 1–100% across studies. Studies were conducted in clinical (59%; *n* = 13), community (36%; *n* = 8) and care (5%; *n* = 1) settings, and in urban (50%; *n* = 11), rural (23%; *n* = 5), both urban and rural (9%; *n* = 2), and unspecified (23%; *n* = 4) environments.

**Table 1:** Demographic and geographical characteristics of all instrumental activities of daily living tools (*n* = 19) included in the review

IADL Tool	Study	Country	Setting	Language of IADL tool	Participant No.	Mean Age	% Female	Education
Thai ADL Scale	Senanarong et al. [50]	Thailand	Clinic, urban	Thai	181	Dementia: 69.51±9.16 Controls: 67.73±9.35	Dementia: 64.8% Controls: 72.7%	Dementia: 0–4 y: 50.28% > 12 y: 11.9% Controls: 0–4 y: 31.82% > 12 y: 26.4%
FAQ-BR/PFAQ	Jomar et al. [20]	Brazil	Community, urban	Portuguese	265	Elderly: 74–84: 44.2% Informants: 75+: 36.6%	Elderly: 74% Informants: 82.1%	≥9 y Elderly: 45.7% Informants: 85.7%
	Aprahamian et al. [22]	Brazil	Clinic, urban	Portuguese	106	AD: 80.28 Controls: 77.95	71.70%	100% illiterate
	Sanchez et al. [21]	Brazil	Community, Urban	Portuguese	68	58±12.9	79.40%	> 9 y: 75%
ADLQ-SV	Gleichgerrcht et al. [12]	Argentina	Clinic, urban	Spanish	40	AD: 79±5.9 bvFTD: 75.4±11 Other: 76.6±8.9	AD: 66% bvFTD: 60% Other: 76%	AD: 12.2±4.7 y bvFTD: 12.9±3.7 y Other: 12.6±4.1 y
ADLQ-BR	Fransen et al. [13]	Brazil	Clinic, urban	Portuguese	90	Controls: 68.07±5.57 MCI: 69.34±7.04 AD: 75.07±6.65	Controls: 74.1% MCI: 71.4% AD: 78.6%	Controls: 14.19±5.57 y MCI: 10.26±4.60 y AD: 6.71±5.16 y
EASI	Pandav et al. [27]	India	Community, rural	Not specified	632	66.5±7.6	46.90%	73.3% illiterate
	Fillenbaum et al. [26]	India	Community, rural	Not specified	387	55–64: 123 participants 65–74: 145 participants 75+: 119 participants	47%	78% illiterate
CSADL	Noroozian et al. [32]	Iran	Clinic, unspecified	Persian	277	Not stated	55%	Male: 9 y Female: 5 y
DADS-Turkish	Tozlu et al. [16]	Turkey	Clinic, unspecified	Turkish	157	77.7±6.8	63.70%	31.8% illiterate
DADS-BR	Bahia et al. [17]	Brazil	Clinic, urban	Portuguese	129	AD: 76.4±6.9 Controls: 74.5±7.3	AD: 64% Controls: 57.5	AD: 6.4±5.1 y Controls: 6.5±4.9 y
IADL-E	Mathuranath et al. [31]	India	Clinic, urban, rural	Not specified	240	67.8±10.5	45%	Dementia: 9.9±4.9 y Controls: 8.9±5.8 y
CHIF	Hendrie et al. [30]	Nigeria/USA	Community, rural	Yoruba/ English	Nigeria: 295 USA: 155	Nigeria: Dementia: 82.9±10.7 Without Dementia: 78.2±6.6 USA: Dementia: 83.4±6.8 Without Dementia: 80.7±6.4	Nigeria: Dementia: 86.8% Without Dementia: 73.9% USA: Dementia: 75% Without Dementia: 70.4%	Nigeria Dementia 0% Without dementia: 13.6% USA Dementia: 8.9±2.5 Without dementia: 9.4±3.0
CA-DFI	Edjolo et al. [29]	Central African Republic/ Republic of Congo	Community, urban, rural	“local languages”	301	76.1±7.4	94%	99.7% Low educational level
IDEA-IADL	Collingwood et al. [8]	Tanzania	Community, rural	Swahili	449 Grouped by IDEA Cognitive Scale scores: ≤7: 40 8–9: 57 ≥10: 352	IDEA Cognitive score levels: ≤7: 80 (IQR: 73.75–85.5) 8–9: 76(IQR: 70–81.25) ≥10: 72 (IQR: 67–79)	IDEA Cognitive score levels: ≤7: 85% 8–9: 71.9% ≥10: 50.6%	Not specified
	Stone et al. [10]	Tanzania	Community, rural	Swahili	Baseline: 153 Follow-up: 98	Baseline: 21.6% 65–69 22.9% 70–74 20.9% 75–79 20.3% 80–84 14.4% 85+ Follow up 15.3 % 65–69 17.3% 70–74 15.3% 75–79 28.6% 80–84 23.5% 85+	Baseline: 67.3% female Follow up: 66.3% female	Without formal education: Baseline: 33.3% Follow up: 29.6%
IDEA-IADL Short	Stone et al. [10]	Tanzania	Community, rural	Swahili	As previous	As previous	As previous	As previous
ADCDS-ADL Turkish	Aysun et al. [24]	Turkey	Clinic, unspecified	Turkish	73	AD: 72.56±10.55 Controls: 68.38±8.82	AD: 56.3% Controls: 58.1%	5.16±3.83 y
ADCDS-ADL Brazil	Cintra et al. [25]	Brazil	Clinic, urban	Portuguese	95	75.9±7.6	60%	Controls: 5.7±4.4 y MCI: 5.2±3.9 y AD: 3.6±3.3 y
GADLS	Paula et al. [34]	Brazil	Clinic, urban	Portuguese	178	MCI <75: 67.04±4.53 MCI 75+: 81.17±5.1 AD <75: 68.97±4.13 AD 75+: 79.47±3.40	Not specified	MCI <75: 5.15±4.29 y MCI 75+: 3.92±3.40 y Dementia <75: 4.68±3.92 y Dementia 75+: 5.26±3.61 y
DAFS-R	Pereira et al. [23]	Brazil	Clinic, urban	Portuguese	89	73.8±6.7	AD: 58% MCI: 74% Controls: 75%	10.3±6 y
	Fransen et al. [13]	Brazil	Clinic, urban	Portuguese	As previous	As previous	As previous	As previous
LBI	Marra et al. [33]	Brazil	Clinic, urban	Portuguese	90	75.46±7.66	75.50%	No education: 24.4% 1–7 y: 56.6% 8 + y: 18.8%
PI	Marra et al. [33]	Brazil	Clinic, urban	Portuguese	As previous	As previous	As previous	As previous
Bristol ADL	Umayal et al. [44]	Sri Lanka	Care	Sinhalese	70	>75: 47.1%	74.30%	≤5 y: 70%
Blessed ADL	Umayal et al. [44]	Sri Lanka	Care	Sinhalese	As previous	As previous	As previous	As previous

ADL, activities of daily living; FAQ, Functional activities questionnaire; BR, Brazil; PFAQ, Portuguese Functional Activities Questionnaire; ADLQ, Activities of daily living questionnaire; SV, Spanish Version; EASI, Everyday Activities Scale –India; CSADL, Cleveland Scale of Activities of Daily Living; DADS, Disability Assessment for Dementia; IADL, Instrumental activities of daily living for elderly people; CHIF, Clinician Home-based Interview to assess Function; CA-DFI, Central Africa Daily Functioning Interference Scale; IDEA-IADL, IDEA study Instrumental Activities of Daily Living Questionnaire; ADCDS-ADL, Alzheimer’s Disease Co-operative Study –Activities of Daily Living Scale; GADLS, General Activities of Daily Living Scale; DAFS-R, Revised Direct Assessment of Functional Status; LBI, Lawton Brody Index; PI, Pfeffer Index; AD, Alzheimer’s disease; MCI, mild cognitive impairment.

Nineteen IADL tools were identified and categorized into three types: translated (*n* = 6), translated and adapted (*n* = 6), and newly developed for the target population (*n* = 7). Results relating to reliability, validity and diagnostic accuracy for all tools can be found in Table 3. Seven discrete IADL tools were assessed by multiple studies and synthesized data for these will be presented below.

**Table 3:** Key results relating to reliability, validity, and diagnostic accuracy of instrumental activities of daily living tools (*n* = 19) in low to middle income countries

IADL Tool	Study	Dementia Criteria	% Dementia/CI	No of items	Total Score	Type of IADL tool	Method	Reliability	Validity	Diagnostic Accuracy/ Criterion Validity
Thai ADL Scale	Senanarong et al. [50]	DSM-IV	88%	13	26	Newly developed for target population	Collected from informants	Inter-rater (*n* = 30): Evaluation 1 ICC: 0.96 (95% CI: 0.91–0.98) Evaluation 2 ICC: 0.93 Test-retest: Rater 1 ICC: 0.92 (95% CI: 0.83–0.96) Rater 2 ICC: 0.89 (95% CI: 0.78–0.95)	Discriminative: Scores: CDR 2 > CDR 1 > CDR 0.5 > CDR 0 Construct: Significant association between each item and the Thai MSE (*r* = 0.69) and CDR (*r* = 0.81) Convergent: Controlling for cognition, correlations between Thai ADL and Barthel Index (*r* = 0.64) and FAQ (*r* = 0.30) remain.
FAQ-BR/PFAQ	Jomar et al. [20]	DSM-IV	43%	10	30	Translated and adapted	Collected from informants		Concurrent: FAQ BR negatively correlated with MMSE (*r* = 0.624, *p* < 0.001) and positively with IQCODE-BR (*r* = 0.755, *p* < 0.001).	Cut off:≥14/30 Sensitivity: 80% (CI: 71.5–86.9) Specificity: 72 (CI: 64.1–79.0) AUC: 79.7% (IC: 74.3% –84.4) PPV: 68.7% (CI: 60.1–76.4) –96/115 people NPV: 82.4% (CI: 74.8–88.5) –49/150
	Aprahamian et al. [22]	DMS-IV, NINCDS-ADRDA	62%						Discriminative: PFAQ significantly different between AD and controls (*p* < 0.001).	Cut off: 11.5 Sensitivity: 85.3 Specificity: 76.5 AUC: 86.4% (SE: 4.3%; 95% CI: 78–94.9%)
	Sanchez et al. [21]	Not used	100% with MMSE < 27, dementia not specified					Cronbach’s alpha: 0.95 Test-retest: ICC: 0.97
ADLQ -SV	Gleichgerrcht et al. [12]	NINCDS-ADRDA: AD McKeith: LBD Lund and Manchester: bvFTD NINDS-AIREN: VaD Benson et al: PCA	100%	28	100	Translated	Collected from informants –based on observation	Cronbach’s alpha for all factors: 0.82–0.96 Inter-rater: Cohen’s K: 0.90 Test-Retest: *r* = 0.95, *p* < 0.001	Concurrent Validity: Correlation with FAQ total (*r* = 0.67, *p* < 0.001) and CDR (*r* = 0.54, *p* < 0.001).
ADLQ-BR	Fransen et al. [13]	AD: Frota et al., 2011 MCI: Winblad et al., 2004	Dementia: 31% MCI: 39%	28	100	Translated	Based on observation	Cronbachs alpha = 0.759	Construct: Correlation between ADLQ-BR and DAFS-R (rho = 0.743).	Controls versus MCI Cut-off 1/100 Sensitivity: 66% Specificity: 69% AUC: 65.3% MCI versus AD Cut off: 21/100 Sensitivity: 93% Specificity: 91% AUC: 97.7%
EASI	Pandav et al. [27]	DSM-III	1%	11	11	Newly developed for target population	Collected from informants			Cut off≥3/11 Dementia versus Controls Sensitivity: 62.5% Specificity: 89.7% AUC: 88.4% PPV: 24.4% NPV: 97.8%
Fillenbaum et al. [26]	Based on Hindi Mental State Examination Scores	Not specified						Cronbach’s alpha: 0.82 Inter-rater reliability: 100% agreement Test-retest: 82–100% agreement	Discriminative and Construct: Differences between Hindi Mental State Examination Stages for EASI (*p* < 0.001).
CSADL	Noroozian et al. [32]	Expert opinion	85%	48	138	Translated	Collected from informants		Discriminative: CSADL Scores: Dementia + AD > MCI	Cognitive impairment versus controls Full scale Cut off: 20 Sensitivity: 90% Specificity: 93% Cut off: 26 Sensitivity: 87% Specificity: 100% IADL Scale Cut off: 20 Sensitivity: 91% Specificity: 100%
DADS-Turkish	Tozlu et al. [16]	DSM-IV, NINCDS-ADRDA	100%	40	100	Translated	Collected from informants	Cronbach’s alpha: 0.942 Inter-rater: ICC: 0.994 (95% CI: 0.987–0.997) Test-retest: ICC: 0.996 (95% CI: 0.991–0.998)	Discriminative: Significant differences for DAD scores between GDS stages: Stage 4 > Stage 5 > Stage 6 + 7. No difference between stages 6 and 7 Construct: Correlation between DAD and Lawton IADL Scale (*r* = 0.928, *p* < 0.001). Convergent: Correlation between MMSE and DADS (*r* = 0.812, *p* < 0.001), DADS and GDS (*r* = 0.880, *p* < 0.001.)
DADS-BR	Bahia et al. [17]		69%	40	100	Translated	Collected from informants	Cronbach’s alpha: 0.77	Convergent: Correlation between DADS and MMSE scores (*r* = 0.044, *p* < 0.001) Scores lower in AD than controls (*p* < 0.01)	AUC: 99.3% Cut-off: 94.6 Sensitivity: 96.6% Specificity: 100 PPV: 100 NPV: 93 Cut-off: 90 Sensitivity: 90% Specificity: 100 PPV: 100 NPV: 81.6 Cut-off: 85 Sensitivity: 81.8% Specificity: 100 PPV: 100 NPV: 71.4
IADL -E	Mathuranath et al. [31]	DSM-IV. AD: NINCDS-ADRDA VaD: NINDS-AIREN	44%	11	22	Newly developed for target population	Collected from informants	Ibadan Results: Cronbach’s alpha: 0.83 Inter-rater: *r* = 0.87, *p* < 0.001	Convergent: IADL-CDI correlated with MMSE (co-efficient: 0.31) –increasing when MMSE increased and vice versa. Construct: IADL-E correlated with DSM-IV (*r* = 0.89), CDR (*r* = 0.82), MMSE (*r* = 0.74) and ACE (*r* = 0.60)	Only cognitive sub score used. Cut off: 16/22 Dementia versus Controls Sensitivity: 91% Specificity: 99% AUC: 97% (94–99) PPV: 0.76%
CHIF	Hendrie et al. [30]	ICD-10, DSM-III AD: NINCDS-ADRDA	Nigeria: 13% USA: 26%	10	20	Newly developed for target population	Clinician interview		Discriminative: Participants without dementia performed better on CHIF than with dementia (*p* < 0.001) Construct: Correlation between CHIF and Blessed Dementia Scale (*r* = 0.56, *p* < 0.001) and MMSE (*r* = 0.44, *p* < 0.001)	Dementia versus Controls AUC: 92.5% Cut off: 18/20 Sensitivity: 89.5% Specificity: 68.5% Cut off: 17/20 Sensitivity: 68.4% Specificity: 82.5%
CA-DFI	Edjolo et al. [29]	DSM-IV AD: NINCDS-ADRDA MCI: Peterson’s Criteria	Dementia: 26.6% MCI: 20.3%	10	Unknown	Newly developed for target population	Collected from informants	Cronbach’s alpha: 0.92	Convergent: 10 item CADFI correlated with walking speed (*r* = 0.431) and CDR (*r* = 0.62) Construct: Item response theory showed <ASK STELLA>	Cognitive Impairment versus Controls Based only on laundry score. Cut off: 0.35 Sensitivity: 96% Specificity: 69% AUC: 87.8% (83.9–91.6)
IDEA-IADL	Collingwood et al. [8]	DSM-IV	26.90%	11	33	Newly developed for target population	Collected from informants	Cronbach’s alpha: 0.959	Criterion: Dementia diagnosis a significant predictor of IADL score Construct: Factor analysis revealed only one factor with eigenvalue > 1, explaining 71.6% of variance.	Dementia versus Controls AUC: 89.6% (CI: 84.2–95.1)
	Stone et al. [10]	DMS-IV	Baseline: 25% Follow-up: 36.7%					Cronbach’s alpha: 0.956		Dementia versus controls Baseline AUC: 90.3% (CI: 85.2–95.3) Follow-up AUC: 62.5% (CI: 50.8–74.2)
IDEA-IADL Short	Stone et al. [10]	As previous	As previous	3	6	Newly developed for target population	Collected from informants		Construct: Factor analysis revealed 2 factors as most strongly predicting dementia.	Baseline AUC: 99.5% (82.0–94.9) Follow up AUC: 62.1% (50.2–73.9) Criterion: Significantly predicted dementia with regression co-efficient: 0.868 (*p* < 0.001)
ADCDS-ADL Turkish	Aysun et al. [24]	NINCDS-ADRDA	44%	23	78	Translated	Collected from informants	Cronbach’s alpha: 0.938 Test-Retest: ICC: 0.998 (95% CI: 0.997–0.999)	Discriminative: ADCS-ADL Scores for CDR Stages 0.5 > 1>2 > 3 Construct: ADSC-ADL highly correlated with BADL (rho = 0.826) and IADL scores (rho = 0.826) on the Modified OARS Convergent: ADCDS-ADL scores are highly correlated with CDR (*r* = 0.828), GDS (*r* = 0.743), but not ADAS Cog (*r* = 0.191)
ADCDS-ADL Brazil	Cintra et al. [25]	AD: NINCDS-ADRDA MCI: Albert and Peterson Criteria	Dementia: 35% MCI: 34%	23	79	Translated and adapted	Collected from informants	Cronbach’s alpha: 0.89	Discriminative: Controls had better ADCDS = ADL scores than MCI and AD (*p* < 0.001). Subitem scores were also better in controls for advanced (*p* < 0.001), IADL (*p* < 0.001) and BADL (*p* = 0.004). Convergent: Association between ADCS-ADL and clinical/neuropsychological diagnosis (ROC = 0.89, *p* < 0.001).	Full scale Cut off: 71/79 Cognitive Impairment versus Controls Sensitivity: 86.2% Specificity: 70% AUC: 81.1% PPV: 86.2% NPV: 70% AD versus Controls Sensitivity: 97% Specificity: 70% AUC: 84.1% PPV: 78% NPV: 95.4% MCI versus Controls Sensitivity: 75% Specificity: 70% AUC: 72.6% PPV: 72.7%
										NPV: 72.4% MCI versus AD Sensitivity: 97% Specificity: 25% AUC: 61.5% PPV: 42.9% NPV: 88.9% IADL Scale Cut-off: 32 Cognitive Impairment versus Controls Sensitivity: 81.5% Specificity: 76.7% AUC: 80% PPV: 88.3% NPV: 65.7% AD versus Controls Sensitivity: 93.9% Specificity: 76.7% AUC: 85.7% PPV: 81.6% NPV: 92% MCI versus Controls Sensitivity: 68.8% Specificity: 76.7% AUC: 72.6% PPV: 75.9% NPV: 69.7% AD versus MCI Sensitivity: 93.9% Specificity: 31.3% AUC: 63.1% PPV: 41.5% NPV: 83.3%
GADLS	Paula et al. [34]	AD: NINCDS-ADRDA MCI: Peterson Criteria	Dementia: 52% MCI: 48%	13	28	Translated and adapted	Collected from informants	Cronbach’s alpha: 0.849		Young MCI versus Young AD (≤74) Sensitivity: 69% Specificity: 62% AUC: 72.5% (CI: 59.9–81.8) Old MCI versus Old AD (>74) Sensitivity: 81% Specificity: 79% AUC: 86.2% (78.1–94.4)
DAFS-R	Pereira et al. [23]	DSM-IV AD: NINCDS-ADRDA MCI: Peterson’s Criteria	Dementia: 29% MCI: 35%	23	##	Translated and adapted	Simulation observed by clinicians	Cronbach’s alpha: 0.78 Inter-rater: ICC: 1–0.918 for all items Test-Retest: ICC: 1–0.915 for all items	Discriminative: Subitems Time Orientation and Communication Scores: MCI + Controls > AD. Subitems Finances and Shopping scores: Controls > MCI > AD. Convergent: Correlation between DAFS and IQCODE (*r* = 0.65, *p* < 0.001). No correlation between DAFS and IQ-CODE when groups examined separately.	AD versus Controls:Cut-off: 86 Sensitivity: 100% Specificity: 93.7% AUC: 99.8% MCI versus Controls: Cut-off: 93 Sensitivity: 80.60% Specificity: 84.4% AUC: 86.8%
	Fransen et al. [13]	As previous	As previous						Construct: Correlation between ADLQ-BR and DAFS-R (rho = 0.743).	Controls versus MCI Cut off: 91/105 Sensitivity: 68% Specificity: 63% AUC: 72.6% MCI versus AD Cut off: 70/105 Sensitivity: 89% Specificity: 83% AUC: 90.5%
LBI	Marra et al. [33]	DSM-IV	100%	8	8 for women 5 for men	Translated	Collected from informants		Construct: Negative correlation found between PI and LBI for full sample (*p* < 0.0001, rho = 0.818) - when looking in each severity - mild (*p* = 0.007, rho = 0.530), severe (*p* < 0.0001, *r* = 0.0723) - in moderate dementia, the questionnaires were not correlated. Discriminative: All dementia severity groups different for LBI scores (*p* < 0.001)
PI	Marra et al. [33]	As previous	As previous	10	30	Translated	Collected from informants		Construct: Negative correlation found between PI and LBI for full sample (*p* < 0.0001, rho = 0.818) - when looking in each severity - mild (*p* = 0.007, rho = 0.530), severe (*p* < 0.0001, *r* = 0.0723) - in moderate dementia, the questionnaires were not correlated. Discriminative: All dementia severity groups different for PI scores (*p* < 0.001)
Bristol ADL	Umayal et al. [44]	ICD-10NA	44%	14	42	Translated and adapted	Collected from informants			Cut-off: 20 Sensitivity: 100% Specificity: 74.2% AUC: 93.3% (95% CI: 87.1–99.5%)
Blessed CERAD	Umayal et al. [44]	As previous	As previous	13	19	Translated and adapted	Collected from informants			Cut-off: 10.5 Sensitivity: 100% Specificity: 89.2% AUC: 89.2% (95% CI: 81.6–96.7%)

ADL, activities of daily living; FAQ, Functional activities questionnaire; BR, Brazil, PFAQ, Portuguese Functional Activities Questionnaire; ADLQ, Activities of daily living questionnaire; SV, Spanish Version; EASI, Everyday Activities Scale –India; CSADL, Cleveland Scale of Activities of Daily Living; DADS, Disability Assessment for Dementia; IADL, Instrumental activities of daily living for elderly people; CHIF, Clinician Home-based Interview to assess Function; CA-DFI, Central Africa Daily Functioning Interference Scale; IDEA-IADL, IDEA study Instrumental Activities of Daily Living Questionnaire; ADCDS-ADL, Alzheimer’s Disease Co-operative Study –Activities of Daily Living Scale; GADLS, General Activities of Daily Living Scale; DAFS-R, Revised Direct Assessment of Functional Status; LBI, Lawton Brody Index; PI, Pfeffer Index; AD, Alzheimer’s disease; MCI, mild cognitive impairment; AUC, area under the curve; PPV, positive predictive value; NPV, negative predictive value.

### Quality assessment

Eleven of the studies included diagnostic accuracy measures and where therefore assessed for quality using the QUADAS 2. Most studies demonstrated some risk of bias; scores are presented in Table 2. All studies were included in the review regardless of the assessed quality to demonstrate the full available data related to the IADL tools assessed within the current literature.

**Table 2:** Consensus scores for the QUADAS-2 demonstrating quality of all diagnostic accuracy studies (*n* = 11) included in this review

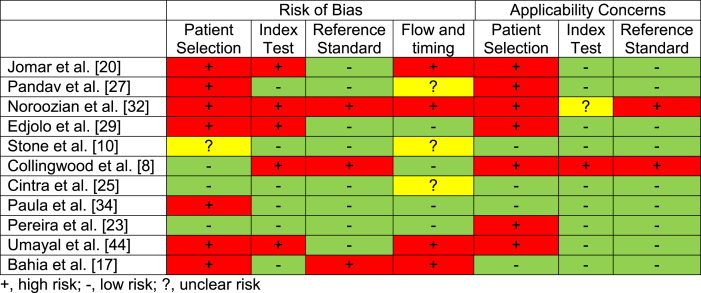

### Translated high-income country developed IADL tools in LMICs

#### Activities of daily living questionnaire (ADL-Q)

The ADL-Q assesses both BADLs and IADLs, evaluating 28 items across six domains: social interaction, social participation, planning/organizing, intellectual activities, feeding, and self-care [11]. This scale is based on an observer’s report, whereby the observer rates the individual’s abilities on a scale of 0–3; higher scores reflect greater impairment. A response option “don’t know/has never done” is also available, and if selected, the item is excluded from the total score. Scores from discrete items are summed to form subdomain scores, and then transformed into a percentage score. No/mild impairment is classified as 0–33%, moderate impairment is 34–66%, and severe impairment is 67–100%

Two studies assessed the use of the ADL-Q, translated into Spanish and Portuguese and conducted in Argentina [12] and Brazil [13], respectively. Both studies took place in clinical settings and urban environments. For Gleichgerrcht et al. [12], 100% of participants had a diagnosis of dementia, and for Fransen et al. [13], 31% had Alzheimer’s disease and 39% had mild cognitive impairment (MCI). On average, people with dementia had 12–13 years of education in Gleichgerrcht et al. [12], while they had 6.7 years in Fransen et al. [13]’s study. Reliability and validity findings are described in Table 3.

Fransen et al. [13] examined diagnostic accuracy of the ADL-Q for detecting MCI compared to normal aging, and for distinguishing Alzheimer’s disease from MCI. With a cut-off of 1%, MCI could be distinguished from controls with 66% sensitivity and 69% specificity (AUC: 0.653; based on Winblad et al. [14]), and with a cut-off of 21%, MCI could be differentiated from Alzheimer’s disease with 93% sensitivity and 91% specificity (AUC: 0.977; based on Frota et al. [15]).

#### Disability assessment for dementia scale (DADS)

The DADS is an informant-based scale which assesses both BADLs and IADLs, evaluating 40 items (17 basic, 23 instrumental) across ten domains. BADL domains include hygiene, dressing, continence, and eating, while IADL domains involve meal preparation, telephoning, going on an outing, finance, and correspondence, taking medication, leisure activities, and housework. Response to each item is yes (1 point) or no (0 points), with the total score ranging from 0–100. Total scores are calculated by summing the score of each item and a percentage is calculated by excluding not applicable answers (e.g., does not do this activity). Lower scores reflect greater impairments in ADLs.

Two studies assessed the use of DADS, translated into Turkish and Portuguese and conducted in Turkey [16] and Brazil [17], respectively. Both studies took place in clinical settings with Bahia et al. [17] reporting an urban environment. Tozlu et al. [16] included 100% of participants with dementia, whereby 31.8% were illiterate. Bahia et al. [17] reported 69% of participants to have dementia, with a mean of 6.4–6.5 years of education.

Diagnostic accuracy was only investigated for DAD-Brazilian version (AUC: 0.993 [17]). With a cut off of 94.6%, dementia could be distinguished from controls with a sensitivity of 94.6%, specificity of 100%, a positive predictive value of 100%, and a negative predictive value of 93%(based on [18, 19]; alternative cut-offs provided in Table 3).

### Translated and adapted IADL tools in LMICs

#### Functional activities questionnaire (FAQ)

The FAQ is an informant-based IADL scale with 10 items: finances, handling documents, shopping, games/hobbies, making tea/coffee, preparing a balanced meal, paying attention/understanding/discussing a tv program/book/magazine, keeping track of current affairs, remembering appointments/occasions/medication, and travelling. Every item is rated between 0–3, with higher scores reflecting greater impairment. If activities are not usually completed by the individual, informants specify whether the individual would be able to carry out the activity. The maximum score is 30.

Three studies examined the FAQ [20–22]. All studies were based in Brazil and used Portuguese versions of the scale. Transcultural adaptions of the FAQ for Brazil were designed, which included reviewing and adapting items and expressions to increase relevance to Brazilian culture. All studies took place in urban environments, with two in community settings [20, 21] and one in clinic [22]. Within each sample, dementia accounted for 43% [20] and 62% [22] of participants. Sanchez et al. [21] did not characterize people with dementia, but all those included had a MMSE score of < 27. For Sanchez et al. [21] and Jomar et al. [20], 75% and 85.7% of informants had 9 + years of education, while the sample in Aprahamian et al. [22] was 100% illiterate.

Both Jomar et al. [20] (AUC: 0.797) and [22] (AUC: 0.864) provided diagnostic accuracy measures. Jomar et al. [20] reported a sensitivity of 80%, specificity of 72%, positive predictive value of 68.7%, and negative predictive value of 82.4% with a cut-off score of 14. Aprahamian et al. [22] used a cut-off of 11.5, showing a sensitivity of 85.3% and specificity of 76.5%

#### Direct assessment of function scale (DAFS)

The DAFS is an observation-based scale which includes BADLs and IADLs. It requires approximately 25 minutes to administer and involves simulating 23 daily tasks across seven domains: time orientation, communication, transportation, finance, shopping, grooming, and eating. The maximum score is 106, with lower scores reflecting greater impairments in ADLs.

Two studies examined DAFS in clinical urban settings in Brazil [13, 23]. The scale was translated into Portuguese and revised to improve relevance for Brazilian culture. For example, currency and stimulus cards with phone numbers and addresses were adapted to Brazilian standards. In Fransen et al. [13], 31% of participants had Alzheimer’s disease and 39% had MCI, while Pereira et al. [23] included 29% of participants with dementia and 35% with MCI. On average, people with dementia had 6.7 years of education in Fransen et al. [13], and 10.3 years in Pereira et al. [23].

Only Pereira et al. [23] reported diagnostic accuracy between dementia and controls (AUC: 0.998, based on [15]). Using a cut-off of 86, DAFS showed a sensitivity of 100% and specificity of 93.7%. Diagnostic accuracy for discriminating MCI from controls and Alzheimer’s disease can be found in Table 3.

#### Alzheimer’s disease cooperative study–activities of daily living scale (ADCS-ADLS)

The ADCS-ADLS is a 23-item informant-based scale which includes assessments of BADLs (6 items), IADLs (10 items), and advanced ADLs (7 items). Each item is rated as either dependent, partially independent, or totally independent, with a maximum score of 79 points, where lower scores reflect greater impairments. It requires approximately 12 minutes to administer.

Two studies assessed ADCS-ADLs, translating it into Turkish and Portuguese and conducted in Turkey [24] and Brazil [25], respectively. For the Turkish version, only minor adjustments to wording were made. For the Brazilian version, an expert committee applied changes to the format of questions, cultural expressions, and vocabulary, and added one sub-item. This adapted ADCS-ADLS Brazilian version was tested in community dwellers with and without cognitive impairment, which led to the removal of “selecting/choosing clothes” and modification of “eating with knives and forks” to “eating independently”. People with dementia encompassed 44% of Aysun et al. [24]’s sample, and 35% of Cintra et al. [25]’s sample with an additional 34% MCI. Mean education ranged from 3.6–5.7 years across the samples.

Cintra et al. [25] reported diagnostic accuracy measures for the Brazilian ADCS-ADLS. Using a cut-off score of 71, dementia could be distinguished from controls with 97% sensitivity, 70% specificity, 78% positive predictive value, and 95.4% negative predictive value (AUC: 0.841, based on [19]). Table 3 provides values for distinguishing controls from overall cognitive impairment and MCI, and for differentiating MCI from dementia.

### Newly developed IADL tools in LMICs

#### Everyday abilities scale for India (EASI)

The EASI is an 11-item informant-based scale involving BADLs and IADLs across four domains: personal care, mobility, social interaction, and cognitive function. A point is scored for each item where impairments are reported, with higher scores reflecting greater impairments. The EASI was developed for a largely illiterate rural Indian population, involving consolation with professional experts, village leaders, and field workers familiar with the community. Items were selected based on activities older adults are culturally expected to carry out, regardless of social status (e.g., wrap/tie lower garments appropriately, express opinions in important family matters).

Two studies assessed EASI in community-based rural settings in India [26, 27]. In Pandav et al. [27], 1% of participants had a dementia diagnosis, while this information was not specified in Fillenbaum et al. [26]. In both studies, there were high levels of illiteracy (73–78%).

Pandav et al. [27] reported diagnostic accuracy measures (AUC: 0.884, based on DSM-III criteria) for distinguishing dementia from controls. Using a cut-off of 3, sensitivity was 62.5%, specificity 89.7%, positive predictive value 24.4%, and negative predictive value 97.8%

#### IDEA-instrumental activities of daily living scale (IDEA-IADL)

The IDEA-IADL is an 11-item informant-based scale assessing IADLs. It can be administered by local healthcare workers to caregivers or relevant informants. It was developed through consultation with district enumerators and local healthcare workers who had extensive training on dementia. Activities that would be expected of an older person, regardless of gender or physical/sensory impairments, were identified (e.g., settle conflicts, preside over ceremonies), resulting 12 relevant activities heavily weighted toward social functions. Following pilot work, one activity was removed (“They make their will and testament and make decisions about their property when they are gone”) as administrators felt uncomfortable asking this. Each item had a four-point scale (0–3) with higher scores reflecting greater impairments. The maximum score is 33.

Two studies examined the IDEA-IADL in community-based rural Tanzania [10, 28]. Collingwood et al. [8] reported 26.9% of participants with a diagnosis of dementia, while in the longitudinal study by Stone et al. [10] had 25% with dementia at baseline (*n* = 153), and 36.7% at follow-up (*n* = 98). Additionally, 33.3% of participants at baseline and 29.6% at follow-up had no formal education; education and literacy rates were not specified in Collingwood et al. [8].

Both studies reported area under the curve scores for accuracy of distinguishing dementia from controls, based on American Psychiatric Association [18] criteria, ranging from 0.625 (follow-up, [10]), 0.896 [8], and 0.903 (baseline, [10]).

## DISCUSSION

In terms of reliability, validity, and accuracy, we highlighted the seven IADL tools which were reported by multiple studies, giving them stronger evidence bases to potentially identify dementia in LMICs, describing their key characteristics (domains, time commitment, scoring process); how they have been developed, translated or adapted; and their accuracy at distinguishing cognitive impairment from normal ageing. We now critically discuss the practical implications of this review in terms of clinical practice and future research.

### Choosing an IADL tool: key considerations

Our findings demonstrate three different categories of IADL tools validated in LMICs. These include translated, translated and adapted, and those newly developed for target populations (i.e., national or regional populations within specific countries). In addition, there were geographical trends in the selection of IADL tools assessed. In African and South Asian LMICs, bespoke culturally-specific tools were predominately investigated [8, 10, 26, 27, 29–31], while translated and adapted tools were mainly used in South America and West Asian LMICs [12, 13, 16, 20–25, 32–34]. This made synthesis of results difficult. Diagnostic accuracy appeared highest in translated/translated and adapted tools, but these findings cannot be readily generalized to African and South Asian LMICs due to cultural differences. For example, while most included LMICs have cultural expectations whereby younger family members assist older members with BADLs when significant disability is present [35], studies based in Africa and South Asia placed significant emphasis on social IADLs (e.g., presiding over ceremonies, keeping up with local affairs/festivals) as younger family members have responsibility over more traditional IADLs measured in Western scales (e.g., financial management) [10, 29]. It is difficult to compare the efficacy of tools which used direct translations of IADL scales used in high-income countries (i.e., translated) and tools which used a cross-cultural adaption process (i.e., translated and adapted). These tools were generally used in Brazil and Turkey, which may hold similarities with the cultures that the tools were originally developed for. This highlights the necessity of first understanding cultural expectations of the target population when choosing an IADL tool, as it should include relevant activities for older adults within that culture to ensure sensitivity for detecting dementia-related impairments [3].

The influence of gender norms and literacy rates are another key consideration when selecting an IADL tool. Most included studies had a predominantly female sample. While this likely reflects the higher prevalence of dementia in women compared to men [36], this limits our understanding of the suitability of IADL tools for men within LMICs. For example, IADL tools with a significant weighting on household activities may not reflect subtle impairments in men within LMICs, as traditional gender roles within most societies dictate that older women predominately carry out household activities (e.g., cooking, cleaning), while men may mainly perform management activities (e.g., keeping financial records) [37]. To account for this, the Lawton Brody Index provided discrete scoring systems for men and women [33] and the IADL-E has an equal number of male- and female-dominant items [31]. An alternative way to negate gender bias is to focus on social IADLs, which both older men and women within the community commonly carry out, such as giving advice [10].

Additionally, low literacy and education rates significantly impact dementia screening and may introduce performance differences across the spectrum of literacy [22]. Articles included in this review similarly highlight significant rates of illiteracy and low educational levels [22, 26, 27, 29, 30]. These illiteracy and education rates can be considered barriers to comprehensive cognitive assessment, and as such, brief cognitive assessments and IADL tools are recommended to reduce bias [38]. Both translated and bespoke IADL questionnaires assessed in populations with high illiteracy and low education demonstrated excellent diagnostic accuracy scores [22, 27, 29], showing that evaluation of the sensitivity and specificity of cut-off IADL scores have been established for illiterate populations in LMICs. Furthermore, Hendrie et al. [30] reported the use of an observational IADL tool (i.e., CHIF) in a Nigerian population with less than four years of education which reported excellent accuracy for identifying cognitive impairment. Ensuring selected IADL tools accommodate for gender or literacy bias is vital to capture cognitively driven impairments early in the disease course.

A final consideration for the selection of IADL tools is the time and expertise required to conduct the assessment. This review describes tools which utilize data collection through informant report, informant interview and direct observation. Informants may provide inaccurate answers to IADL questions due to their perception of the “normal” aging process or the stigma surrounding cognitive impairment [10]. Direct observation is generally considered the gold standard of IADL assessment, demonstrated by the excellent diagnostic accuracy scores reported [12, 13, 23]. However, such tools require significant staff training, time, and resources which may be inappropriate for wide-scale dementia screening in LMICs. The WHO mhGAP (Mental Health Gap Action Programme) proposes that community health workers could deliver interventions and diagnostic services, with basic training. Thus the most appropriate tool for dementia screening in LMICs may be short, simple to score IADL questionnaires, based on informant report, tailored for use in community settings [3]. In four studies, where IADL assessments were carried out by community/field workers, good diagnostic accuracy and inter-rater reliability were found [10, 26–28]. However, Stone et al. [10] found significant discrepancy in diagnostic accuracy values in a longitudinal follow up, with baseline scores showing excellent accuracy for identifying dementia (AUC: 0.99–0.90) and one year follow-up indicating poor accuracy (AUC: 0.625). Baseline assessment was conducted by a skilled health-care team while longitudinal follow-up was carried out by village enumerators. It is proposed that discrepancies were due to subjectivity in interpreting answers provided to the questions introduced by village enumerators. This highlights the importance of appropriate assessor training and selecting IADL tools which do not require a high dependency on individual judgement in the grading process, such as dichotomous scales (e.g., “yes/no”).

### Strengths and limitations of this review

A significant strength of this review was our comprehensive and rigorous search strategy (see Supplementary Material) and use of multiple electronic databases to identify potential articles for inclusion. We also hand-searched reference lists of all included articles to maximize the scope of our search. We carried out independent title, abstract, and full-text screening and all disagreements were adjudicated by a third reviewer. Our quality assessment indicated that, although most diagnostic accuracy studies included demonstrated some risk of bias, overall, they showed moderate-good quality. However, we only included articles available in English due to limited resources and may not have captured all relevant IADL tools for LMICs. For example, we have limited representation of Asian countries despite significant work reported on cognitive assessments in Asia [39]. Additionally, we excluded studies which combined IADL questions with cognitive assessments within one tool (e.g., Everyday Cognition Scale [40]) as they did not fall within the strict remit of our review question. These tools could also be considered within the diagnostic process in LMICs, and further investigation should determine how useful they may be. A variety of IADL tools were assessed within this review across a diverse range of populations. As such, a meta-analysis was inappropriate to conduct at this time but may be useful in the future when greater evidence bases are built for discrete measures. At this time, the evidence for any tool is limited by inconsistencies in validation methods, and lack of external validation across all scales. As such, we do not recommend any particular IADL tool as a diagnostic aid for dementia in LMICs but do provide suggestions to bridge this gap.

### Recommendations for future research

A significant gap identified by this review is the lack of research around the generalizability of IADL tools, both across LMICs and within LMICs, as illustrated by the seven newly developed tools across six LMICs included in this review. Their item domains are similar; for example, both the EASI and the IDEA-IADL consider variations in ability to be involved in family matters and to take part in festivals and ceremonies [8, 10, 26, 27]. However, there has been no investigation into the feasibility of using bespoke IADL tools created for a specific LMIC in LMICs that hold similar cultural ideals. In contrast, there is significant evidence that tools which have been translated and adapted from Western high-income countries are feasible and acceptable to use in South America. For example, the FAQ shows acceptable-excellent diagnostic accuracy in Brazil [20–22] and is one of the most commonly used IADL scales worldwide [41]. This lends more confidence to the generalizability of translated scales on a global scale, but these tools have not been investigated in Africa or South Asian countries which may have unique cultural expectations, as discussed previously. Therefore, we recommend that already existing tools—either translated from Western high-income countries or bespoke tools developed for LMICs (e.g., EASI, IDEA-IADL) be considered and evaluated for use before new scales are developed for specific target populations.

Additionally, within LMICs there is limited understanding of how transferable IADL tools of all types are between urban and rural communities. For example, most translated tools investigated in South America were applied in clinical urban environments and required skilled professionals to conduct the assessments, which may not be applicable or feasible for rural communities. In contrast, Edjolo et al. [29] highlights that items included in the CA-DFI may not be applicable to urban settings, such as assessing one’s ability to work in fields. As such, suitable urban alternatives need to be validated. Only two studies explicitly included both urban and rural communities, highlighting a significant gap that should be addressed through future studies [29, 31].

A major limitation to the current state of research is the lack of external validation of IADL tools within LMICs. Most studies included in this review involved scale development or initial validation. For the majority, reliability and technical validity were established, whereby IADL tools showed acceptable internal consistency, inter/intra-rater reliability, and associations with other measures of cognitive impairment (e.g., cognitive scales). However, without external validity, findings of each IADL tool cannot be generalized to communities beyond those investigated or to individuals who present in a different way (e.g., prodromal dementia). This is particularly relevant to newly developed tools for target populations as translated tools have generally demonstrated good validity in populations from different backgrounds and cultures, such as the FAQ [20–22, 41–43]. Several studies also excluded people with physical impairments or other neurological conditions [12, 13, 16, 17, 22–25, 34, 44], limiting our understanding of how IADL tools might distinguish dementia from other disorders in a population-level cohort. The validity of IADL tools could also be strengthened by establishing their relationship with recognized objective gold-standard biomarkers, such as blood tests and neuroimaging [45]. While this may not be standard clinical practice in LMICs due to the expensive nature and resource-intensity of these biomarkers, it would improve confidence for clinicians to apply these simple IADL tools as diagnostic benchmarks. Ideally, further technical, and external validity within a population sample should be established before wide-scale adoption of an IADL tool within a LMIC.

### Implications for practice

Due to limited financial and healthcare resources within LMICs, it is vital to establish simple, sensitive dementia screening and diagnostic tools to promote early detection [3]. Timely diagnosis allows individuals and their families to better understand the diagnosis, consider appropriate care and treatment plans and avail of non-pharmacological interventions and drug therapies early in the disease [46]. Beyond clinical use, early and accurate diagnosis is important for researchers and policymakers to identify the true prevalence of dementia in LMICs and develop appropriate action plans for global dementia strategies. Additionally, IADL tools could support both clinicians and researchers by identifying changes in function due to disease progression and determining care needs of an individual. This review has indicated that IADL tools which are culturally appropriate and applicable to settings of different language, education and healthcare resources can be implemented in LMIC settings with good-excellent accuracy for distinguishing dementia from normal ageing. It is important to acknowledge, however, that there is no “perfect” measure; diagnostic practice generally requires a variety of tools to support clinical decision-making. It is recommended that IADL tools are used in combination with at least one brief global cognitive assessment [3], such as translated versions of the Mini-Mental State Examination or culturally-tailored assessments such as the IDEA Cognitive screen [10, 28, 39]. This combination can strengthen the accuracy of the diagnostic battery. For example, Pandav et al. [27] reported the highest paired sensitivity (90.6%) and specificity (68.2%) when the EASI was coupled with a comprehensive cognitive battery. Similarly, Stone et al. [10] found that the combination of both the IDEA-IADL and the IDEA cognitive screen showed the highest accuracy for distinguishing cognitive impairment from normal aging (AUC: 0.93) compared to single measures (AUC: 0.84–0.89). These measures could be supported by inexpensive digital markers, such as measures collected from wearable technology (e.g., gait, sleep), which are culturally-naïve [47]. Such devices have been found to be acceptable and feasible to use in older adults in LMICs, as conducted by community field workers [48] and are considered useful supportive markers for dementia diagnosis in high-income settings [49]. Further work is needed to 1) validate their utility in the LMIC diagnostic pathway and 2) identify which combination of diagnostic tools provides the greatest sensitivity and specificity for identifying dementia in culturally-diverse LMIC settings.

## CONCLUSION

This review synthesized the current literature on the reliability, validity, and accuracy of IADL tools for identifying dementia in LMICs. From our findings, we present the seven IADL tools with the strongest evidence base. We also highlight key considerations for choosing an IADL tool for use in a LMIC, such as selecting tools that are culturally appropriate, account for bias introduced by gender-roles and literacy rates, easy and quick to use and which can be conducted by any volunteer with the right training. There are significant gaps in the research which must be addressed, including greater technical validity against established gold-standard biomarkers of dementia and external validation of IADL tools within different regions, populations, cultures and across LMICs. Future work should consider combinations of diagnostic markers, such as IADL tools, brief cognitive assessments, and novel measures such as those derived from digital technology, to establish the most appropriate and sensitive diagnostic toolkit for dementia in LMICs.

## Supplementary Material

Supplementary Material
