# Supplementary material for: Instrumental Activities of Daily Living Scales to Detect Cognitive Impairment and Dementia in Low- and Middle-Income Countries: A Systematic Review
Source: J Alzheimers Dis. 2021 Aug 31;83(1):451–74. doi: 10.3233/JAD-210532 (PMC8461665; doi:10.3233/JAD-210532)
Supplement: Supplementary Material [file jad-83-jad210532-s001.pdf]

Supplementary Material

Instrumental Activities of Daily Living Scales to Detect Cognitive Impairment and Dementia in Low- and Middle-Income Countries: A Systematic Review

This file contains the terms that were used in our search strategy.

Combinations of the following terms were searched across the databases: dementia, cognitive impairment, Alzheimer*, cognitive dysfunction, cognitive deficit, cognitive decline, neurocognitive disorder, instrumental activities of daily living, IADL, complex activities of daily living, extended activities of daily living, extended ADL, complex ADL, activities of daily living, ADL, daily activities, functional ability, functional disability, daily functioning, functional assessment, independent living, developing, developed, less* developed, under developed, underdeveloped, middle income, low* income, countr*, nation, population, world, economy, economies, low* gdp, low* gnp, low* gross domestic, low* gross national, LMIC, LMICs, third world, LAMI, transitional, Africa, Asia, Caribbean, West Indies, South America, Latin America, Central America, Afghanistan, Albania, Algeria, American Samoa, Angola, Argentina, Armenia, Azerbaijan, Bangladesh, Belarus, Belize, Benin, Bhutan, Bolivia, Bosnia and Herzegovina, Botswana, Brazil, Bulgaria, Burkina Faso, Burundi, Cabo Verde, Cape Verde, Cambodia, Cameroon, Central African Republic, Chad, China, Colombia, Comoros, Congo, Costa Rica, Cote d'Ivoire, Cuba, Djibouti, Dominica, Dominican Republic, Ecuador, Egypt, El Salvador, Equatorial Guinea, Eritrea, Eswatini, Swaziland, Ethiopia, Fiji, Gabon, Gambia, Georgia, Ghana, Grenada, Guatemala, Guinea, Guinea-Bissau, Guyana, Haiti, Honduras, India, Indonesia, Iran, Iraq, Jamaica, Jordan, Kazakhstan, Kenya, Kiribati, Korea, Kosovo, Kyrgyz Republic, Lao, Lebanon, Lesotho, Liberia, Libya, Madagascar, Malawi, Malaysia, Maldives, Mali, Marshall Islands, Mauritania, Mauritius, Mexico, Micronesia, Moldova, Mongolia, Montenegro, Morocco, Mozambique, Myanmar, Burma, Namibia, Nauru, Nepal, Nicaragua, Niger, Nigeria, North Macedonia, Pakistan, Papua New Guinea, Paraguay, Peru, Philippines, Romania, Russian Federation, Russia, Rwanda, Samoa, "Sao Tome and Principe", Senegal, Serbia, Sierra Leone, Solomon Islands, Somalia, South Africa, South Sudan, Sri Lanka, St Lucia, St Vincent and the Grenadines, Sudan, Suriname, Syrian Arab Republic, Syria, Tajikistan, Tanzania, Thailand, Timor-Leste, Togo, Tonga, Tunisia, Turkey, Turkmenistan, Tuvalu, Uganda, Ukraine, Uzbekistan, Vanuatu, Venezuela, Vietnam, West Bank and Gaza, Yemen, Zambia, Zimbabwe.
